# Mater-Bi/Brewers’ Spent Grain Biocomposites—Novel Approach to Plant-Based Waste Filler Treatment by Highly Efficient Thermomechanical and Chemical Methods

**DOI:** 10.3390/ma15207099

**Published:** 2022-10-12

**Authors:** Aleksander Hejna, Mateusz Barczewski, Paulina Kosmela, Olga Mysiukiewicz, Paweł Sulima, Jerzy Andrzej Przyborowski, Daria Kowalkowska-Zedler

**Affiliations:** 1 Institute of Materials Technology, Poznan University of Technology, Piotrowo 3, 61-138 Poznań, Poland; 2 Department of Polymer Technology, Gdańsk University of Technology, Narutowicza 11/12, 80-233 Gdańsk, Poland; 3 Department of Genetics, Plant Breeding and Bioresource Engineering, University of Warmia and Mazury in Olsztyn, Plac Łódzki 3, 10-724 Olsztyn, Poland; 4 Department of Inorganic Chemistry, Gdańsk University of Technology, Narutowicza 11/12, 80-233 Gdańsk, Poland

**Keywords:** Mater-Bi, brewers’ spent grain, biocomposites, structure-property relationships, filler modification, recycling

## Abstract

Thermoplastic starch (TPS) is a homogenous material prepared from native starch and water or other plasticizers subjected to mixing at a temperature exceeding starch gelatinization temperature. It shows major drawbacks like high moisture sensitivity, poor mechanical properties, and thermal stability. To overcome these drawbacks without significant cost increase, TPS could be blended with bio-based or biodegradable polymers and filled with plant-based fillers, beneficially waste-based, like brewers’ spent grain (BSG), the main brewing by-product. Filler modifications are often required to enhance the compatibility of such composites. Herein, we investigated the impact of BSG thermomechanical and chemical treatments on the structure, physical, thermal, and rheological performance of Mater-Bi-based composites. Thermomechanical modifications enhanced matrix thermal stability under oxidative conditions delaying degradation onset by 33 °C. Moreover, BSG enhanced the crystallization of the polybutylene adipate terephthalate (PBAT) fraction of Mater-Bi, potentially improving mechanical properties and shortening processing time. BSG chemical treatment with isophorone diisocyanate improved the processing properties of the composites, expressed by a 33% rise in melt flow index. Depending on the waste filler’s selected treatment, processing, and rheological performance, thermal stability or interfacial adhesion of composites could be enhanced. Moreover, the appearance of the final materials could be adjusted by filler selection.

## 1. Introduction

Among the currently most popular trends in polymer science is reducing the environmental impact of polymer technology [1,2,3]. Over the last decades, researchers have aimed to develop bio-based and biodegradable materials that could substitute conventional polymers [4,5]. Among the most popular solutions offered on the industrial scale are biobased polymers like poly(lactic acid) (PLA) or polyhydroxyalkanoates (PHAs) or biodegradable petroleum-based polymers like poly(ε-caprolactone) (PCL) or polybutylene adipate terephthalate (PBAT) [6,7,8,9,10]. However, due to the complex manufacturing process and relatively small production scale, their price is not competitive compared to conventional plastics [11]. Therefore, research and industrial environments seek other, more cost-effective solutions for manufacturing bio-based sustainable polymer materials. Among them are blends with biopolymers, mainly starch, which due to its low cost and hardly limited availability, is an auspicious solution for manufacturing sustainable materials [12,13,14,15]. However, to be processed by conventional methods applied in polymer technology requires proper modifications, notably the conversion to thermoplastic starch (TPS), which significantly reduces its melting point [13,16,17,18]. Due to its biodegradability and availability, TPS gained much attention as potential packaging material [19,20,21,22]. Nevertheless, despite the extensive research over the last few years, it shows significant drawbacks. One of them is fragility and low water resistance, limiting the range of TPS applications [23]. Others, seemingly unexpected, are related to the environmental impact, particularly water and land use, which are higher than conventional plastics [24]. Therefore, despite the economic advantages of TPS over polymers like PLA, PHA, PCL, or PBAT, it is beneficial to blend TPS with other polymers to overcome these issues. Such solutions are currently offered by multiple companies, including Novamont SpA (various Mater-Bi grades), JinHui Zhaolong (Ecowill), Biotec (Bioplast), AGRANA Beteiligungs-AG (AGENACOMP), or Biome Bioplastics Limited (Biome).

Another solution to popularize bio-based and biodegradable polymers is the introduction of bio-based fillers, which reduce the use of polymers and may enhance their performance. Potential candidates are materials of different origins, chemical compositions, physical structures, or morphology [25,26]. The desired composite performance should drive the proper selection of filler. For more advanced applications requiring, e.g., superior mechanical performance or biocompatibility, purer materials like cellulose nanocrystals, nanofibrillated cellulose, or bacterial cellulose can be applied [27,28,29]. However, different waste-based fillers seem more viable for more common materials, particularly disposable ones, whose price and biodegradability are often crucial.

The potential candidates are multiple plant-based wastes from the food and agricultural sectors [30,31,32]. An exciting candidate is the brewers’ spent grain, a significant by-product of the brewing industry. Like other plant-based materials, it is composed mainly of cellulose, hemicellulose, and lignin [33]. Currently, it lacks a broader industrial use despite its high volume of production, which is almost 12 million tonnes globally [34]. Our previous works on PCL-based composites investigated their applications as fillers for polymer composites [35,36,37]. However, similar to the results of other researchers [38,39], insufficient interfacial compatibility between BSG and polymer matrix was noted, mainly when the unmodified filler was applied, which could limit the potential application range of composites. Incorporating thermomechanical treatment improved the composites’ performance because of changes in fillers’ polarity [36,37], suggesting it is a promising direction.

To ensure more substantial improvement, it is beneficial to provide covalent bonding at the interface, which could be provided by the chemical treatment and introduction of functional groups on the fillers’ surface [40]. Such an approach could overcome the drawbacks related to insufficient interfacial adhesion and significantly enhance the performance of composites. The most popular method is applying anhydrides or anhydride-grafted polymers, which can create covalent bonds with hydroxyl groups of plant-based fillers, significantly improving composites’ compatibility [41]. Among the potential modifiers are also the isocyanates, compounds showing high reactivity with multiple functional groups present in the structure of plant-based fillers and bio-based and biodegradable polymers. They can create strong urethane bonds in the reaction with hydroxyl groups [42]. In composites based on conventional matrices like polyolefins, isocyanates reduce the polarity difference, often improving mechanical performance [43,44]. When matrices containing functional groups are applied, covalent bonding can be developed, significantly improving interfacial adhesion, which has been proven for PLA [45], PBAT [46,47], and PCL-based biocomposites [48].

In the presented work, we aimed to examine the combined impact of brewers’ spent grain thermomechanical treatment and chemical modification with isophorone diisocyanate on the structure and performance of biocomposites based on biodegradable Mater-Bi matrix comprising of PBAT, TPS, and PCL. Applied filler treatments were conducted to enhance the interfacial interactions and improve the mechanical performance of biocomposites, which are often affected by the incorporation of plant-based waste fillers.

## 2. Materials and Methods

### 2.1. Materials

The commercial starch-based biomaterial Mater-Bi NF803 from Novamont SPA (Novara, Italy) was applied as a matrix for prepared composites. According to the producer, it was characterized by the value of melt flow index of 3.5 g/10 min (150 °C/5 kg) and melting temperature of 110 °C.

Brewers’ spent grain was obtained from Energetyka Złoczew sp. z o. o. (Knapy, Poland). According to the supplier, it originated from the production of light lager and consisted solely of barley malts. The supplier already dried the applied BSG. The chemical composition of applied BSG is presented in Table 1 and was analyzed in the Department of Genetics, Plant Breeding and Bioresource Engineering at the University of Warmia and Mazury in Olsztyn following the methodology described by Stolarski et al. [49].

Isophorone diisocyanate (IPDI) acquired from Sigma Aldrich (Poznań, Poland) was applied as a chemical modifier of BSG fillers. Its purity was 99%, and it was characterized by a density of 1.06 g/cm^3^.

Distilled water, methanol, acetone, and *n*-pentane were applied as solvents during BSG particle dispersibility tests. All solvents, except water, were purchased from Sigma Aldrich (Poland). Distilled water (H_2_O) was characterized by a relative polarity of 1.000, a density of 0.998 g/cm^3,^ and dynamic viscosity of 0.8921 mPa·s. Next, methanol (CH_3_OH) with a relative polarity of 0.762, a density of 0.792 g/cm^3^, and dynamic viscosity of 0.5480 mPa·s was used. Acetone (CH_3_C=OCH_3_) was characterized by a relative polarity of 0.355, a density of 0.789 g/cm^3,^ and dynamic viscosity of 0.3160 mPa·s. The least polar solvent, *n*-pentane (CH_3_(CH_2_)_3_CH_3_), was characterized by a relative polarity of 0.009, a density of 0.626 g/cm^3,^ and dynamic viscosity of 0.2224 mPa·s.

### 2.2. Modifications of Brewers’ Spent Grain

Before incorporating it into a polymer matrix, BSG was subjected to thermomechanical and chemical treatment. The thermomechanical modification was described in detail in previous works [50,51]. Samples applied in the presented work were modified using an EHP 2x20 Sline co-rotating twin-screw extruder from Zamak Mercator (Skawina, Poland), using a throughput of 3 kg/h and a screw speed of 225 rpm and barrel temperature of 180 or 240 °C.

Moreover, to evaluate the impact of isocyanate modification, the BSG fillers were modified with 5 wt% of IPDI using GMF 106/2 Brabender batch mixer at room temperature (varied from 21.1 to 23.1 °C) and rotor speed of 100 rpm. The proper amount of filler was placed in an internal mixer with a calculated amount of diisocyanate, respectively, to the filler mass. Mixing was performed for 5 min, and samples were put in zipper storage bags. Isocyanate content was based on the results presented in our previous paper [48].

### 2.3. Preparation of Polymer Composites

Composites were prepared using GMF 106/2 Brabender batch mixer at 140 °C and rotor speed of 100 rpm. The processing time equaled 6 min, including the 1-min phase of matrix plasticization and 5 min of melt blending with selected filler. Filler content in each sample was fixed at 30 wt%. Prepared composites were compression molded at 150 °C and 4.9 MPa for 1 min and then kept under pressure at room temperature for another 5 min to solidify the material. Obtained samples were coded as X/Y, where X stands for the BSG thermomechanical treatment temperature and Y for IPDI content applied during modification. Unfilled Materi-Bi was processed similarly for comparison.

### 2.4. Characterization Techniques

The chemical structure of composites was determined using Fourier transform infrared spectroscopy (FTIR) analysis performed by a Nicolet Spectrometer iS50 from Thermo Fisher Scientific (Waltham, MA, USA). The device had an ATR attachment with the Specac Quest single reflection diamond attenuated total reflectance (ATR) accessory. Measurements were performed with 1 cm^−1^ resolution in the range from 4000 to 400 cm^−1^ and 64 scans.

A test for the dispersibility of BSG particles in different solvents was performed to evaluate the changes in the fillers’ polarity. The method was adopted from previous research work [52]. Specimen of BSG weighing approximately 1 g was put in a vial, and 20 mL of proper solvent was added. Then, vials were shaken vigorously for one minute, and the sedimentation of particles was recorded. Photographs were made right after the shaking was stopped, then each minute for five minutes, and after ten minutes. Four solvents were used, water, methanol, acetone, and *n*-pentane, which were selected based on their polarity, expressed by the relative polarity index, according to data presented by Reichardt [53]. 

Scanning electron microscopy (SEM) was performed using the model Tescan MIRA3 microscope. Structure analysis has been performed for brittle fractured polymeric and composite compression molded samples. The JEE 4B vacuum evaporator from Jeol USA (Peabody, MA, USA) was applied to coat the analyzed samples with thin, approx. 20 nm, carbon layer. The measurements were conducted with an accelerated voltage of 5 kV and magnifications of 200× and 5000×.

The samples’ L*, a*, and b* color coordinates were determined using an NR145 colorimeter (Envi Sense, Lublin, Poland) using the 45°/0° geometry. The Browning Index (BI) was calculated according to Equations (1) and (2) [54]:

BI = ((x − 0.31) · 100)/0.17(1)

where:

x = (a* + 1.75 · L*)/(5.645 · L* + a* − 0.3012 · b*)(2)

Other parameters were also calculated from the values of L*, a*, and b* also, other parameters were calculated. Chroma, hue, and total color difference (ΔE) were determined according to Equations (3)–(5):

Chroma = ((a*)^2^ + (b*)^2^)^0.5^ (3)

Hue = arctan(b*/a*)(4)

∆E = ((∆L*)^2^ + (∆a*)^2^ + (∆b*)^2^)^0.5^ (5)

The thermal stability of materials was determined by thermogravimetric analysis (TGA) with the temperature set between 35 °C and 800 °C at a heating rate of 15 °C/min using a TG 209 F1 Netzsch (Selb, Germany) apparatus. Samples of 10.0 ± 0.1 mg and ceramic pans were applied. TGA analysis was performed under a nitrogen and oxygen atmosphere to analyze fillers’ antioxidant potential comprehensively.

The differential scanning calorimetry (DSC) analysis was conducted to evaluate the thermal performance of Mater-Bi and its composites filled with BSG. The 204 F1 Phoenix apparatus from Netzsch (Selb, Germany) was used for the analysis. The parameters of analysis were as follows: temperature range from −80 to 170 °C; heating rate—15 °C/min; atmosphere—nitrogen, weight of samples—5.0 ± 0.2 mg; crucibles—aluminum with pierced lids.

The Zwick (Ulm, Germany) mFlow plastometer was applied to evaluate the melt flowability of Mater-Bi and its composites filled with BSG. Analysis was performed following ASTM D1238 standard at 170 °C with 2.16 kg load. Melt flowability was quantitatively expressed as mass or volume melt flow index (MFI).

To evaluate the rheological performance of analyzed materials, Anton Paar (Graz, Austria) MCR 301 rotational rheometer was used. Analysis was carried out in the oscillatory mode, at 170 °C, with 1 mm gap between 25 mm parallel plates. Prior to the dynamic measurements in the frequency sweep mode, samples were analyzed in strain sweep experiments, which were also performed at 170 °C, at a constant angular frequency of 10 rad/s in the varying strain window of 0.001–100%. Such an approach enables determination of 0.05% strain value, which is further applied for dynamic frequency sweep measurements. The angular frequency used during the studies was in the range of 0.05–500 rad/s.

## 3. Results

### 3.1. Chemical Structure of Applied Fillers

**Figure 1:**
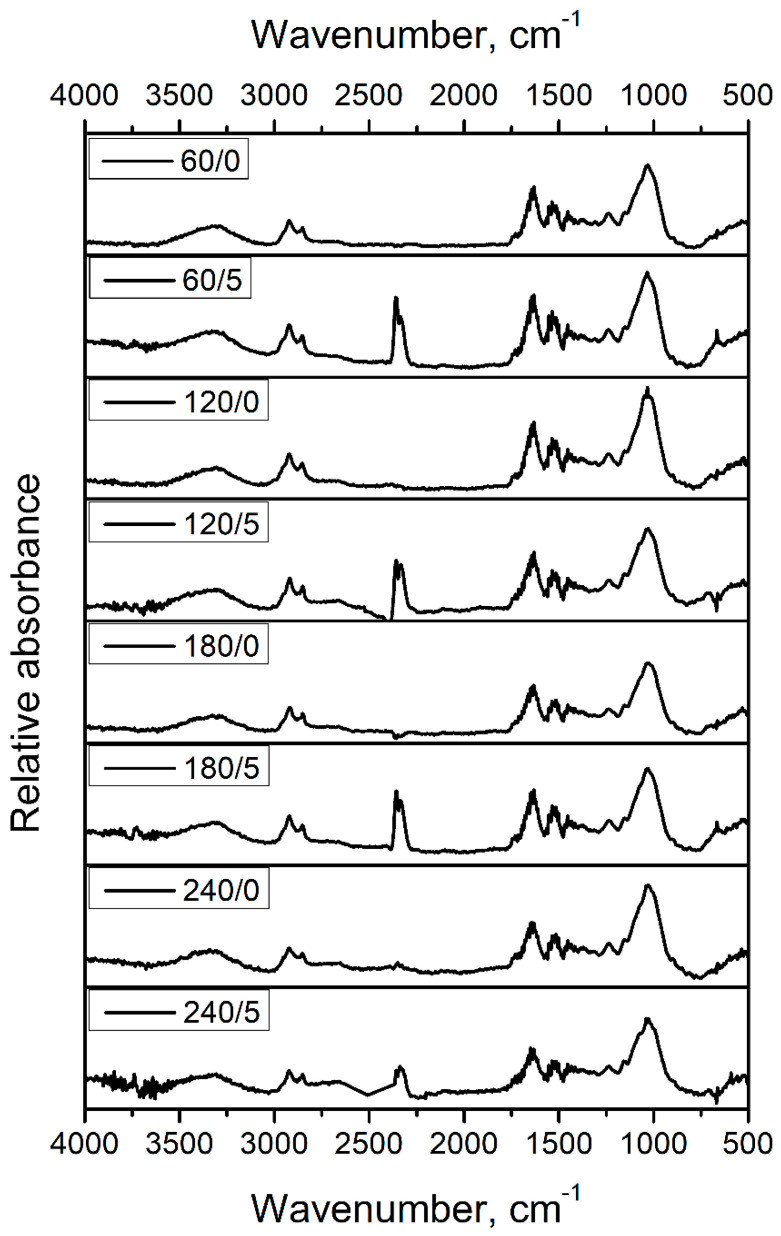
FTIR spectra of different grades of BSG applied as fillers for prepared biocomposites.

Figure 1 presents the FTIR spectra of applied fillers subjected to thermomechanical and chemical treatment. It can be seen that all of the materials show spectra typical for lignocellulose materials. Signals in the range of 3292–3356 cm^−1^ are characteristic of the stretching vibrations of hydroxyl groups, which are widely present in the structure of primary plant-based materials’ components—cellulose, hemicellulose, and lignin [55]. Moreover, slight broadening of these signals for increasing extrusion temperature, and mainly for IPDI modification, may indicate the increase in the number of N-H bonds present in amine and amide groups, whose signals are noted above 3300 cm^−1^ [56]. Such an effect may be related to the Maillard reactions and the generation of urethane bonds between hydroxyls of fillers and isocyanate groups [40]. Peaks typical for the symmetric and asymmetric stretching vibrations of C-H bonds in CH_2_ and CH_3_ end groups were noted at 2854 and 2922 cm^−1^ [57]. Bending vibrations of these bonds caused the presence of a band at 1453 cm^−1^.

Other signals for lignocellulose materials were noted at 1640, 1530, 1243, and 1022 cm^−1^. Their origin is often complex and is related to overlapping multiple particular peaks. For example, a peak at 1640 cm^−1^ is often ascribed to the stretching vibrations of C=C, C=N, and C=O bonds [58]. A peak around 1530 cm^−1^ is attributed to the N-H bending and=C and C-N stretching [59]. Bands around 1243 and 1022 cm^−1^ are typical for stretching vibrations of double and single carbon-oxygen bonds [25]. These bonds are widely present in the structure of BSG components (e.g., lignin or proteins), melanoidins (generated during thermomechanical treatment), and urethane bonds (resulting from the reaction between hydroxyls present in the BSG structure and isocyanate groups of IPDI). Therefore, these signals hardly provide quantitative information about the chemical structure in such complex systems as analyzed fillers.

Moreover, for IPDI-modified fillers, the significant doublet peak at 2335 and 2358 cm^−1^ was noted, related to the stretching vibrations of the unreacted isocyanate group [60]. Its presence can be attributed to the relatively low reactivity of isophorone diisocyanate compared to other, more popular isocyanates, especially aromatic ones like methylene diphenyl or toluene diisocyanates, which was shown in previous work on cellulose filler modifications [52].

To further evaluate the changes in fillers’ chemical structure and polarity, in particular, the sedimentation tests in different solvents were performed. The sedimentation process of suspensions is strongly affected by their concentration, fluid viscosity, interactions between solid particles, density differences between phases, and interfacial fluid-particle interactions [61]. The impact of interfacial interactions increases with the specific surface area of particles, so indirectly also with their diameters, and becomes noticeable for micrometric sizes [62]. Therefore, in the presented case, the changes in the sedimentation behavior between particular BSG fillers can be attributed to the varying hydrophilicity of their surfaces. Table 2 and Table 3 present the impact of brewers’ spent grain thermomechanical and chemical modifications on the stability of their suspensions in solvents characterized by different polarities. In all photographs, the solvents are ordered in the following manner—water, methanol, acetone, and *n*-pentane (from left to right).

It can be seen that the materials thermomechanically modified at 60 and 120 °C create the most stable suspensions at polar solvents, mainly water but also methanol. The sedimentation of particles in acetone and *n*-pentane occurs significantly faster, indicating poor compatibility with these solvents and significant polarity differences. Increasing treatment temperature causes noticeable differences in samples’ behavior, especially at 240 °C, where acetone suspension is noticeably more stable. It points to the polarity reduction, probably caused by the caramelization and Maillard reactions occurring during BSG extrusion. Caramelization involves dehydration and condensation, while Maillard reactions occur between amino and carbonyl groups [63]. As a result, the number of polar groups is reduced and replaced with less polar structures.

Chemical modification with diisocyanate caused visible changes in the fillers’ behavior, especially in contact with water, associated with the chemical reactions between solvent particles and residual, unreacted isocyanate groups. It caused the generation of carbon dioxide and the resulting porous layer on top of the flask. Considering less polar solvents, the behavior was also changed. Before the isocyanate modification, the stability of methanol suspensions was superior to those of acetone, particularly for 60 and 120 °C modifications. The effect was less pronounced at 180 °C. Nevertheless, chemical treatment resulted in similar stability of these suspensions, indicating a decrease in polarity. Considering the values of the methanol and acetone relative polarity (0.762 and 0.355, respectively), similar stability suggests the noticeable reduction of polarity after IPDI treatment. The polarity shift was also observed for the extrusion temperature of 240 °C, even despite the lower polarity prior to the chemical modification. Such an effect is in line with our previous results on the isocyanate modifications of cellulose fibers [52]. Nevertheless, the polarity shift was not so significant as for the neat cellulose due to the lower initial polarity of BSG, which can be associated with the chemical composition, e.g., the presence of lignin or hemicellulose [64]. Moreover, among the tested diisocyanates (hexamethylene, isophorone, methylene diphenyl, and toluene), the isophorone diisocyanate was found to be the least effective in the reduction of cellulose polarity, which was attributed to its aliphatic chemical structure and presence of cyclohexane ring [52].

### 3.2. Structure and Performance of Prepared Composites

**Figure 2:**
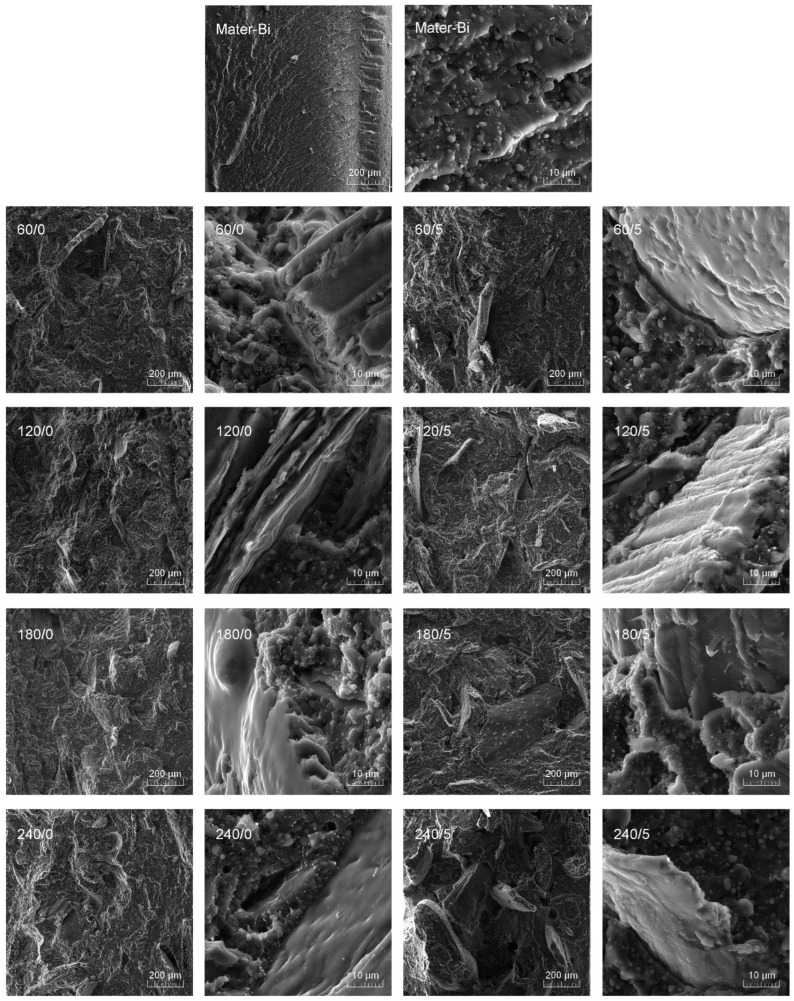
SEM images of Materi-Bi and its composites containing BSG subjected to thermomechanical and chemical treatments; taken with two different magnifications.

Figure 2 presents a summary of SEM images for non-filled Mater-Bi and composite samples made at lower and higher magnifications, which allowed the assessment of the degree of filler dispersion and the fracture structure and the estimation of adhesion changes at the interface. The micrometric spherical inclusions, which can be seen mainly for unmodified Mater-Bi, are associated with the presence of semicrystalline starch granules, which were not efficiently plasticized. A similar effect was noted by Aldas et al. [65].

All composite samples reveal the uniform distribution of the filler in the analyzed area, and a comparable size characterizes the particles. However, it shows fewer pull-out holes after removing the filler on the brittle fractured surface of the composites sample series containing BSG, subjected only to thermomechanical treatment. Moreover, when comparing the SEM images taken with higher magnification for composites containing BSG with different thermal histories, the addition of isocyanate has an adverse effect on interfacial adhesion. For the series made using IPDI, evident gaps in the interfacial area are visible, which proves insufficient adhesion between the filler and the polymer [66]. Such an effect may indicate the excessive reduction of fillers’ polarity resulting from isocyanate treatment.

The results of the spectroscopic color analysis of prepared materials are collected in Table 4. As can be seen, the unfilled Mater-Bi sample is white, with a slight yellowish hue as indicated by the b* value of 9.92). Adding either kind of brewers’ spent grain makes the composite samples much darker (L* in the range of 33.8–39.7) and brown, which was associated with the color of BSG fillers [50]. It can also be seen that the color of the studied samples depends on the processing temperature and modification of the filler with isocyanate. The composites containing the chemically treated BSG are generally darker than those filled with unmodified brewers’ spent grain. However, the difference between them in the case of the highest processing temperature is negligible, which is represented by the decrease of ΔE with thermomechanical treatment temperature. The values of both a* and b* are also higher in the case of the samples with isocyanate-treated filler, meaning that the red and yellow hues are easier to notice. Nevertheless, in the case of both types of samples, the a* and b* values are in the ranges typical for brown colors [25]. These parameters shift toward higher values along with the processing temperature of the filler, but for samples 240/0 and 240/5, hardly any difference is noted, which makes composites’ appearance almost indistinguishable.

The values of the Browning Index change similarly, indicating that the brownest are the samples with the filler processed at 240 °C, pointing to the highest yield of caramelization and Maillard reactions. Chemical treatment of the BSG with isocyanates also causes an increase in BI, and the difference is significantly distinct in the case of the lower processing temperature. This course of changes is understandable, as dark-colored products of the Maillard reactions were generated during the thermomechanical processing of BSG [67]. It is widely known that the browning of substances depends on the Maillard reaction temperature. Therefore, it is reasonable that the composites containing BSG processed at higher temperatures present higher BI values. It can also be noticed that the results obtained for the composite samples are much higher than the Browning Index obtained for the fillers, as reported in our previous research [50]. The additional influence of temperature can explain this difference during the melt mixing of the BSG with Mater-Bi, which also caused the caramelization of the filler and resulted in its further browning. Table 4 also presents the values of chroma and hue, two other parameters describing color. Their changes were similar because they are determined by a* and b* values. Relatively low chroma values point to the low color saturation, typical for grey and brown colors. Moreover, hue values are typical for brown shades, which according to the literature data, are in the range of 30–70° [68].

Generally, it can be seen that the color of prepared composites can be slightly adjusted by the conditions of BSG thermomechanical and chemical modifications. Such an effect should be considered very beneficial for potential applications because, by adjusting of filler treatment and its loading, a broad range of materials’ colors could be obtained without the additional incorporation of pigments and dyes.

**Figure 3:**
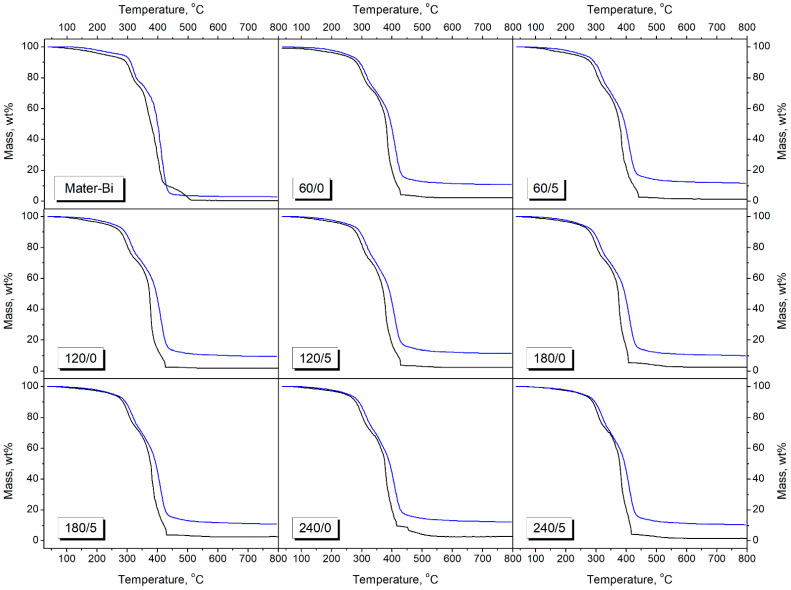
TGA curves registered for Mater-Bi and its composites (upper blue curves registered in N_2_ and lower black curves in O_2_).

**Figure 4:**
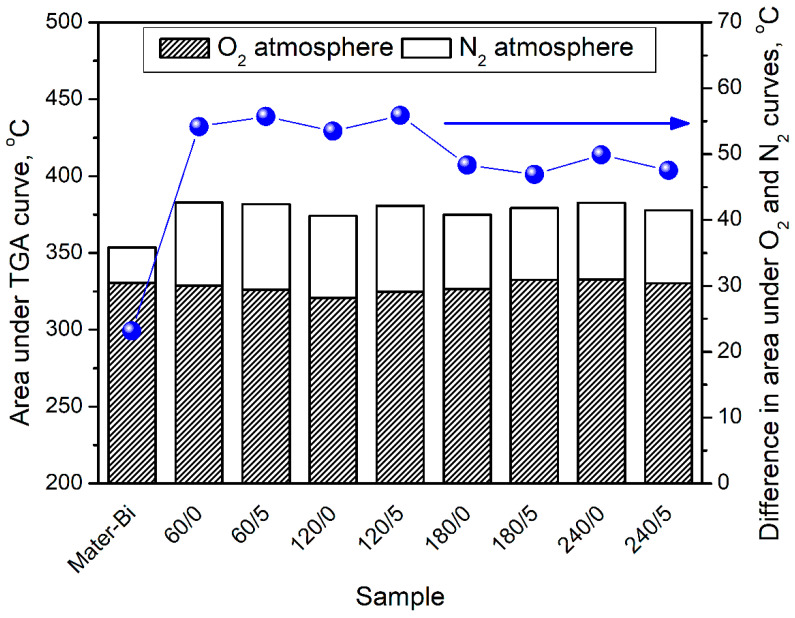
Results of integration of TGA curves registered for Mater-Bi and its composites.

The results of the prepared composites’ thermogravimetric analysis conducted in an inert and oxidative atmosphere are summarized in Table 5 and Figure 3 and Figure 4. It can be seen that the atmosphere of analysis significantly affected its results. For all the analyzed samples, the onset of thermal decomposition, determined as the temperature of 2 wt% mass loss, was noticeably lower in the oxygen atmosphere than in nitrogen. In an inert atmosphere, all samples began to decompose between 190.4 and 197.3 °C irrespective of the composition. However, for the O_2_ atmosphere, the onset was directly increased by the temperature of BSG thermomechanical treatment. Therefore, for the unfilled Mater-Bi matrix, the difference between atmospheres was 54.6 °C, while the introduction of filler significantly reduced it, even to 16.2 °C for the 240/5 sample. Such an effect was attributed to the enhanced oxidative resistance caused by the filler modification resulting in the generation of melanoidins, compounds characterized by potent antioxidant activity [69]. Similar enhancement was noted for the temperatures associated with 5 wt% mass loss. Such results suggest that incorporating thermomechanically modified BSG or other waste plant-based fillers may benefit the thermooxidative resistance of wood-polymer composites.

Table 5 also presents the temperature positions of peaks on differential thermogravimetric curves, which are attributed to the local maximum mass loss. Under nitrogen atmosphere, all samples showed three-step decomposition, characteristic of Mater-Bi NF grades [70,71]. The first peak (T_max1_) was related to the degradation of plasticized starch, particularly glucose rings of amylose and amylopectin [71,72]. The last, most substantial peak (T_max3_) was characteristic of the decomposition of PBAT, the main component of Mater-Bi NF803 material, and PCL [70]. For composite samples, all the peaks were noticeably shifted compared to the unfilled matrix, which is associated with the decomposition of BSG fillers. As presented in previous work [51], DTG curves of thermomechanically treated BSG showed two significant peaks, ~281 and ~342 °C, attributed to the decomposition of hemicelluloses and celluloses [73]. Therefore, due to incorporating 30 wt% of fillers, T_max1_ and T_max2_ were shifted towards lower temperatures. The main peak characteristic for PBAT and PCL decomposition was hardly affected.

Considering oxidative atmosphere, the unfilled matrix showed four main decomposition steps, while composites only two. Additional peak (T_max4_) present for Mater-Bi material was probably related to the thermolysis of lower molecular weight residues of PBAT and PCL from the primary decomposition step attributed to main-chain scissions (T_max3_) [74]. On the other hand, signal T_max2_ was not present for composites, probably due to the overlapping with the main T_max3_ peak characteristic for PBAT and PCL decomposition.

Thermogravimetric analysis in the inert and oxidative atmosphere may provide exciting insights into the oxidative resistance of material [75]. In Figure 4, there are presented sizes of the area obtained by integrating TGA curves from analysis in nitrogen and oxygen. It can be seen that the slightest difference can be noted for unfilled Materi-Bi, which can be related to the course of its decomposition under a nitrogen atmosphere. As shown in the presented study and the works of other researchers [70,71], Mater-Bi decomposes almost entirely under a nitrogen atmosphere. Therefore, even under an oxidative atmosphere, mass loss is not significantly higher, resulting in a relatively small difference between the N_2_ and O_2_ curves. Considering prepared composites, the difference between decomposition under inert and oxidative atmosphere decreases with the rise of BSG thermomechanical treatment temperature. For samples containing BSG modified at 60 and 120 °C, the area below the O_2_ curve accounted for 85.3–85.9% of the area for the N_2_ curve. For modification temperatures of 180 and 240 °C, it increased to 87.0–87.6%, confirming the increased antioxidant activity of BSG resulting from performed modifications, as shown in our previous work [51]. The above-mentioned Maillard reactions result in the generation of melanoidins, compounds characterized by potent antioxidant activity, which may enhance the oxidative resistance of polymer composites [34]. Previous work [29] reported a similar effect when dealing with poly(ε-caprolactone)/brewers’ spent grain composites. Obtained results indicate that although the prepared fillers alone cannot be considered very efficient antioxidants significantly protecting the polymer phase from oxidation, thermomechanical modification of fillers is an auspicious research direction. As a result, the improved antioxidant activity of fillers could slightly reduce the use of synthetic antioxidants, which could be beneficial from environmental and economic points of view.

**Figure 5:**
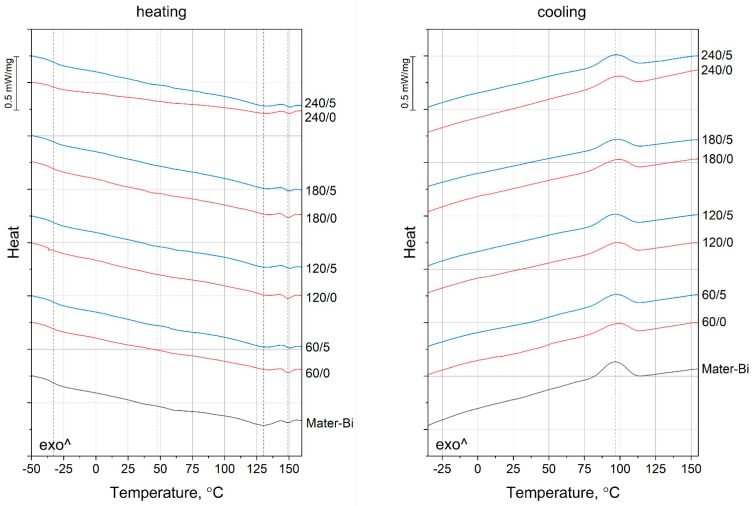
DSC thermograms registered during the second heating and cooling of Mater-Bi and its composites.

Glass transition, crystallization, and melting temperatures determined by DSC are collected in Table 6. The thermograms obtained during the measurement can be found in Figure 5. Mater-Bi, a blend of thermoplastic starch, poly(butylene adipate-co-terephthalate), and polycaprolactone, can be characterized by complex thermal behavior. The inflection visible at the heating curve around −33 °C can be attributed to the glass transition of PBAT. The slightly visible change in the curve run around 47 °C comes from melting the PCL fraction. Melting of PBAT takes place around 130 °C, as indicated by an endothermic peak, followed by melting of the thermoplastic starch around 150 °C [71]. Only one peak can be distinguished on the cooling curve—the exotherm around 96 °C indicates crystallization of poly(butylene adipate-co-terephthalate) [70]. Because of the relatively low intensity of melting peaks and difficulties in separating signals from the different components’ phase transitions, the material’s crystallinity could not be calculated.

The BSG-filled composites show very similar courses of the DSC curves as the matrix material; however, some phase transition temperatures are shifted. In the case of the samples containing the unmodified filler, the glass transition temperature of PBAT is lower than the unfilled Mater-Bi. This result indicates that BSG does not restrict the movements of polymeric macromolecules—on the contrary, it shows a slight plasticizing effect, probably due to the presence of protein. The isocyanate-treated filler has a different influence on T_gPBAT_, which in this case is higher than Mater-Bi. It can be deduced that the chosen chemical treatment method improves the filler and the polymeric matrix interactions. Interestingly, the 240/5 sample shows different behavior, and it undergoes glass transition at a similar temperature to the 240/0 one, indicating lower chemical treatment efficiency. As seen on the FTIR spectra obtained for the different fillers (see Figure 1), all the chemically treated BGS contain isocyanate groups, which can react with the polymer during the melt blending. The intensity of the double peak at 2335 and 2358 cm^−1^ coming from this group in the case of the 240/5 filler is much lower than for the BGS grades processed at lower temperatures. It can be concluded that the IDPI modifier reacted with oxidated filler, so it contains a smaller amount of free isocyanate groups able to interact with the polymeric matrix.

The melting temperature of the PCL fraction also changes due to the addition of different types of filler. In this case, the course of changes is related to the BSG processing conditions or its chemical treatment. As the polycaprolactone content in Mater-Bi is relatively small, the signal of its phase transition is weak and can be easily disturbed by noise. Different behavior can be observed for the PBAT melting peak—in the case of the composite samples, it is up to 5 °C higher than the unfilled Mater-Bi. This result indicates that bigger or more perfect PBAT crystals are created in the presence of the filler, regardless of its type. When we compare this to the increased crystallization temperature of poly(butylene adipate-co-terephthalate) observed in the presence of the filler, it can be stated that the addition of BSG promotes the formation of the crystalline phase of PBAT. Considering that higher T_crPBAT_ were obtained for the samples containing filler without the chemical modification, which can have a plasticizing effect on Mater-Bi, it can be decided that BSG is not a typical nucleating agent but rather facilitates the movement and arrangement of the macromolecules. However, a detailed description of the influence of this filler on crystallization cannot be made without a thorough analysis of crystallization kinetics.

In the case of the melting point of the thermoplastic starch, all the studied samples show similar values. It can be concluded that BSG, regardless of its processing conditions and chemical modification, has the most significant influence on the thermal properties of the PBAT phase of Mater-Bi.

**Figure 6:**
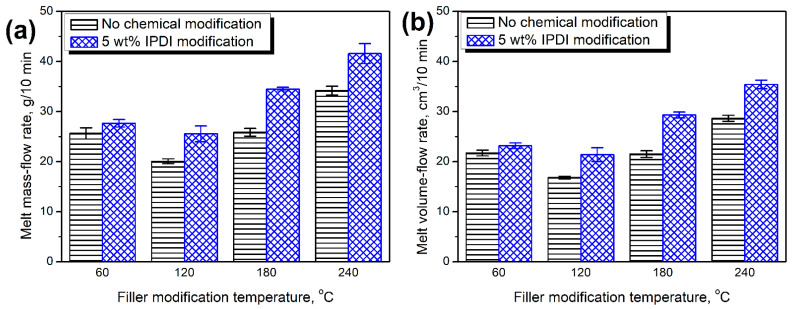
Values of (**a**) melt mass-flow rate and (**b**) melt volume-flow rate measured for different composite samples.

Figure 6 presents the impact of filler modifications on the melt flowability of Mater-Bi-based biocomposites. It can be seen that the increasing thermomechanical treatment temperature improved the flowability of materials. Only for 120 °C, a slight decrease was noted compared to the lower temperature, especially for fillers without additional chemical treatment. Nevertheless, the general trend pointed to the beneficial impact of the filler’s extrusion temperature, which could be attributed to the decreasing particle size, as presented in our previous paper on BSG thermomechanical treatment [51]. Such an effect could facilitate the alignment of particles along the flow direction and increase the melt flow index. Moreover, the flowability was enhanced by the chemical modification of brewers’ spent grain with isophorone diisocyanate, which enhanced the matrix-filler adhesion. Higher values of melt flow index resulting from the enhanced interfacial interactions in polymer composites were also noted by other authors [76].

**Figure 7:**
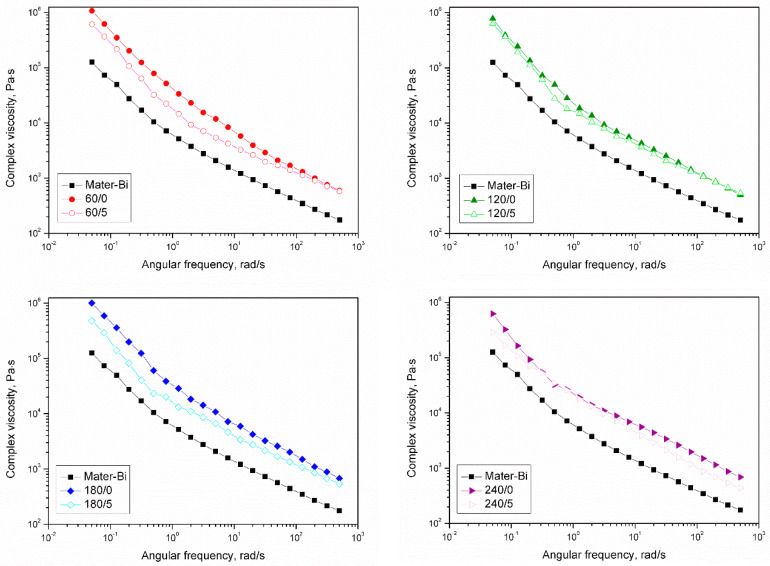
Complex viscosity curves of pure Mater-Bi and Mater-Bi composites filled with BSG subjected to various thermomechanical and chemical treatments.

Figure 7 shows the complex viscosity curves for unmodified Mater-Bi and composites containing organic fillers subjected to thermomechanical and chemical treatment using isophorone diisocyanate. The shape of the curves showing no low values of angular frequency Newtonian plateau is typical for starch-based thermoplastics [77]. While in the case of composite materials, there is no Newtonian behavior that may be related to solid-like rheological behavior resulting from the formation of rigid physical structures in polymeric bulk. Similarly, the unmodified Mater-Bi also showed independent storage modulus behavior (Figure S1—Supporting information); however, the origin of this phenomenon for pure Mater-Bi is the poor miscibility of the polymer system related to dispersion of non-plasticized starch in the polyester matrix [77,78,79]. At the same time, all tested material samples showed shear thinning behavior. 

**Figure 8:**
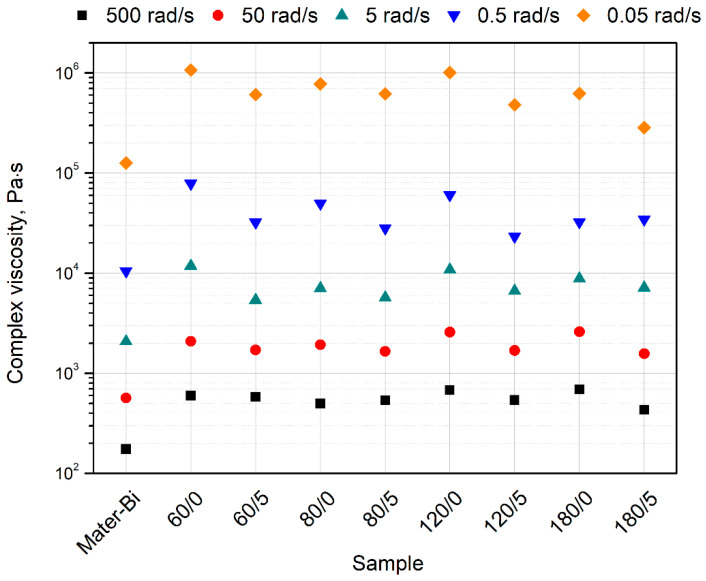
Values of complex viscosity of prepared materials at selected angular frequencies.

Additionally, the values of complex viscosity at selected angular frequencies have been summarized in Figure 8 to illustrate the differences between the tested material series. As can be seen, significant changes in the complex viscosity in the entire tested angular frequency range were recorded only when comparing unfilled Mater-Bi with composites. In the case of the remaining samples, only a clear tendency can be observed between the series of BSG subjected to thermomechanical modification and additional chemical modification. In the case of all series, a decrease in viscosity was noted after the application of diisocyanate during the technological process. These differences increased with increasing angular frequency.

The results of oscillatory measurements are in good agreement with preliminary investigations of processing properties assessed by MFI determination. The addition of all filler types caused an increase in the viscosity; however, the less pronounced influence of isocyanate-modified fillers was noted. For all composite series similar trend may be observed; the composites containing thermomechanically modified BSG exhibit lower values of complex viscosity in the whole considered angular frequency range. It can be supposed that chemical modification reduces the filler’s surface area and improves the compatibility between polymeric matrix and particle-shaped organic inclusions. According to Parcella et al. [80], the lowered viscosity of filled polymeric compositions that shows pseudo-solid-like behavior may be correlated with changes in the interactions of polymeric chains with modified natural filler structure. At the same time, it should be emphasized that while the beneficial effect of isocyanate on the rheological properties of Mater-Bi/BSG composites can be noticed, there was no correlation between the intensity of the impact of IPDI on compatibility depending on the change in process temperature.

In Table 7, the storage (G′), loss (G″) modulus, and complex viscosity values, measured at an angular frequency of 0.05% and 5%, are additionally collectively presented. In the case of data registered at low values of angular frequency, a more significant value G′ from G is observed for all material series (which confirms solid-like rheological behavior or elastic response with infinite relaxation time, as mentioned earlier), filler-to-filler interactions [77,81,82]. What is relevant for composites containing BSG subjected to chemical treatment is that the differences between G ‘and G″ are much lower, suggesting a better filler dispersion in the polymer matrix and reduced effects from surface modification leading to surface modification compatibilization with the polymeric matrix. For measurements taken at 5 rad/s, the composites filled using IPDI-modified BSG showed lower loss modulus values, which may be straightly interpreted as the viscous-dominated behavior of molten composites. The consequent lower complex viscosity values for those series suggest better compatibility. Therefore, when oscillatory rheological results are compared with MFI results, it can be stated that using IPDI for surface treatment of BSG during extrusion improves its compatibility with Mater-Bi, and in effect, lead to improved processability of composites manufacturing with their use.

## 4. Conclusions

The presented paper aimed to investigate the influence of brewers’ spent grain thermomechanical and chemical treatments on the structure, physical, thermal, and rheological performance of composites based on the Mater-Bi NF803 matrix. The reported BSG modification process should be considered auspicious. The chemical structure and surface polarity of modified BSG could be changed by adjusting treatment conditions, which is crucial for providing strong interfacial interactions in polymer composites.

Moreover, thermomechanical treatment of BSG stimulated the occurrence of caramelization and Maillard reactions on the particles’ surface, which resulted in enhanced antioxidant activity of fillers. In composites, such an effect was expressed by shifting thermal decomposition onset under an oxidative atmosphere towards higher temperatures. The shift was proportional to the increase in thermomechanical treatment temperature. This effect should be considered very promising because it may provide additional features to the polymer composites by proper pretreatment of BSG filler and could slightly reduce synthetic antioxidants, which would benefit from environmental and economic points of view. Except for the thermomechanical modifications, additional chemical treatment slightly enhanced thermal stability, suggesting enhanced compatibility. Similar conclusions can be drawn from the results of the DSC analysis. The BSG modification with IPDI improved the interfacial adhesion; however, the modification efficiency is the highest in the case of filler subjected to extrusion at temperatures up to 180 °C. Moreover, the addition of the waste filler showed a beneficial influence on the crystallization of the PBAT fraction of Mater-Bi, which could potentially improve mechanical properties and shorten processing time. At the same time, the BSG treatment with isophorone diisocyanate resulted in improved processing properties of the composites, which was found based on rheological analysis and melt flow index values. Therefore, it is necessary to conduct additional research that will allow for a compromise between the two phenomena listed.

In conclusion, depending on the details of the applied treatment, processing, and rheological performance, thermal stability or interfacial adhesion of composites could be enhanced. Moreover, the appearance of the final materials could be adjusted by filler selection. Further works could include an analysis of the filler loading impact and applying other diisocyanate modifiers, which could broaden the possibility of processing and performance adjustment.

## Figures and Tables

**Table 1:** Chemical composition of applied brewers’ spent grain.

Composition, %_dw_		Element Content, %_dw_	
Cold water extractives	38.49 ± 0.70	C	52.46 ± 0.69
Hot water extractives	43.62 ± 1.11	H	7.88 ± 0.10
Neutral detergent extractives	9.80 ± 0.18	N	3.25 ± 0.02
Cellulose	10.85 ± 0.54	S	0.158 ± 0.001
Hemicellulose	32.65 ± 0.73	Cl	0.057 ± 0.008
Lignin	3.09 ± 0.03		
Ash	3.26 ± 0.04		

**Table 2:** The influence of treatment temperature on BSG behavior in particular solvents (from left to right—water, methanol, acetone, *n*-pentane).

Time, Minutes	Sample			
	60/0	120/0	180/0	240/0
0	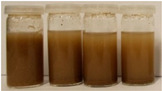	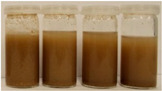	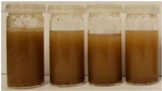	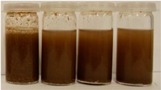
1	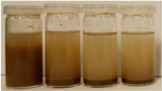	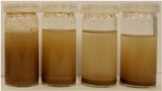	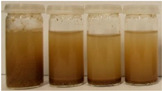	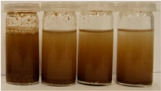
2	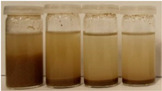	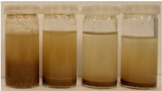	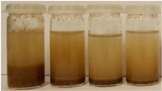	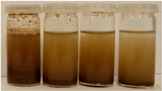
3	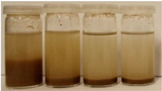	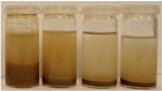	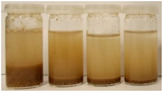	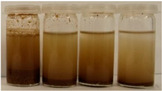
4	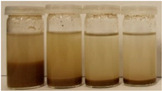	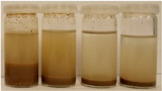	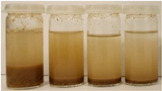	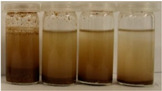
5	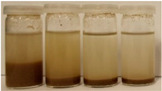	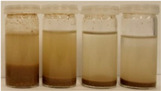	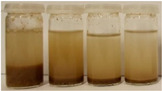	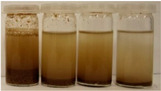
10	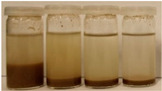	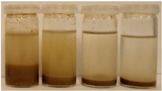	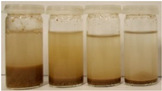	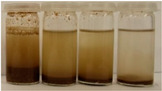

**Table 3:** The influence of the IPDI modification on the behavior of fillers in particular solvents (from left to right—water, methanol, acetone, *n*-pentane).

Time, Minutes	Sample			
	60/5	120/5	180/5	240/5
0	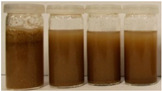	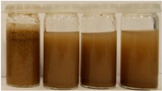	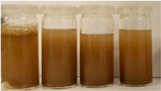	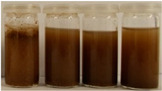
1	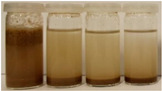	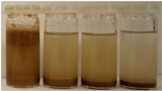	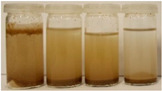	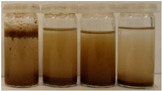
2	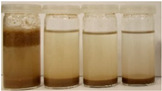	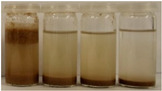	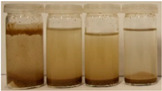	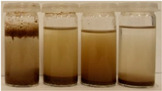
3	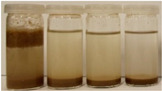	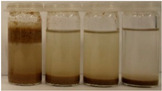	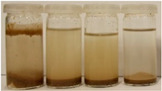	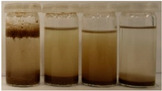
4	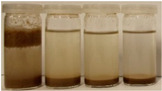	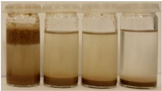	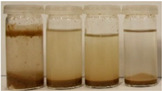	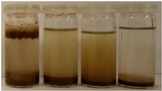
5	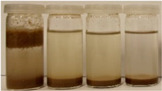	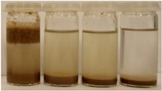	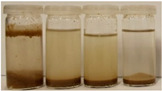	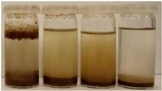
10	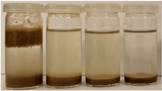	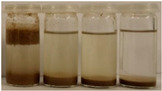	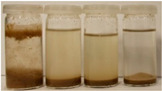	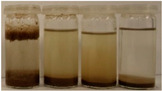

**Table 4:** The color parameters or unfilled Mater-Bi and prepared Mater-Bi/BSG composites.

Sample	Mater-Bi	60/0	60/5	120/0	120/5	180/0	180/5	240/0	240/5
L*	84.54 ± 0.17	39.32 ± 0.40	33.84 ± 0.19	39.10 ± 0.32	37.46 ± 0.11	39.71 ± 0.28	37.25 ± 0.28	34.98 ± 0.10	34.42 ± 0.17
a*	−0.09 ± 0.04	4.62 ± 0.11	8.01 ± 0.06	6.17 ± 0.14	7.50 ± 0.05	9.09 ± 0.18	9.31 ± 0.26	9.66 ± 0.10	9.54 ± 0.17
b*	9.92 ± 0.08	7.47 ± 0.17	11.94 ± 0.12	9.84 ± 0.14	12.02 ± 0.21	14.26 ± 0.35	14.48 ± 0.28	13.62 ± 0.09	13.20 ± 0.17
BI	1.08	10.19	20.00	13.61	17.20	19.54	21.26	23.00	22.99
Chroma	9.92	8.78	14.38	11.61	14.17	16.91	17.21	16.70	16.29
Hue, °	89.5	58.3	56.1	57.9	58.0	57.5	57.3	54.7	54.1
Chemical treatment ΔE	-	-	7.84	-	3.03	-	2.48	-	0.71
Digital color reproduction									
Appearance	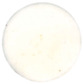	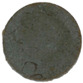	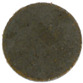	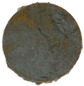	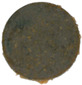	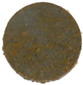	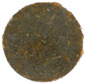	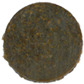	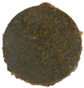

**Table 5:** Results of thermogravimetric analysis of prepared materials.

Sample	Atm.	T_−2%_, °C	T_−5%_, °C	T_−10%_, °C	T_−50%_, °C	T_max1_, °C	T_max2_, °C	T_max3_, °C	T_max4_, °C	Residue, wt%
Mater-Bi	N_2_	197.3	279.5	310.6	399.9	319.6	362.1	412.1	-	2.88
60/0		195.4	253.2	293.0	396.8	314.8	354.4	409.8	-	10.67
60/5		193.6	256.1	294.7	395.3	317.2	355.2	409.7	-	11.68
120/0		193.8	257.1	293.1	396.6	312.1	354.9	412.1	-	9.44
120/5		192.7	256.8	294.8	393.9	314.6	352.7	407.1	-	11.36
180/0		190.4	250.1	291.2	395.2	314.6	356.8	412.1	-	9.99
180/5		192.0	255.2	295.0	394.7	317.2	352.6	409.7	-	10.93
240/0		192.7	255.7	290.8	392.8	312.3	359.7	409.8	-	12.18
240/5		191.8	257.9	296.5	394.8	322.3	351.9	409.8	-	10.45
Mater-Bi	O_2_	142.7	223.3	295.4	378.1	311.8	364.2	401.8	505.3	0.19
60/0		142.4	232.3	282.0	380.1	303.1	-	383.9	-	2.35
60/5		144.9	228.5	282.7	377.8	301.4	-	383.9	-	1.23
120/0		151.7	232.3	278.5	373.8	296.9	-	377.4	-	1.84
120/5		156.2	240.4	281.0	372.4	299.7	-	377.2	-	2.34
180/0		158.1	239.6	280.1	373.6	296.9	-	375.5	-	2.53
180/5		170.4	250.6	286.0	377.3	300.8	-	383.3	-	2.68
240/0		165.1	247.7	280.6	376.6	297.9	-	380.4	-	2.73
240/5		175.6	253.8	288.9	378.3	305.0	-	382.5	-	1.55

**Table 6:** Values of phase transition temperatures obtained for Mater-Bi and its composites.

Sample Name	T_gPBAT_, °C	T_mPCL_, °C	T_mPBAT_, °C	T_mstarch_, °C	T_crPBAT_, °C
Mater-Bi	−32.8	47.4	130.4	149.1	96.4
60/0	−34.1	45.1	135.1	149.3	99.3
60/5	−31.4	44.5	132.9	150.7	97.5
120/0	−36.9	47.3	134.2	148.8	98.5
120/5	−32.0	46.9	133.6	150.6	97.4
180/0	−36.9	49.2	133.4	149.2	99.9
180/5	−31.9	44.1	134.3	150.2	98.1
240/0	−34.8	46.0	132.9	149.9	99.7
240/5	−34.1	47.1	132,9	150,7	97,8

**Table 7:** Storage (G′) and loss (G″) moduli and complex viscosity (η*), measured at angular frequencies of 0.05 and 5 rad/s.

Sample	G′, Pa	G″, Pa	η*, Pa·s	G′, Pa	G″, Pa	η*, Pa·s
	ω = 0.05 rad/s			ω = 5 rad/s		
Mater-Bi	4 220	4 660	126 000	7 590	7 110	2 080
60/0	43 100	31 700	1 070 000	47 100	35 600	11 800
60/5	23 800	19 000	609 000	16 900	21 000	5 390
120/0	28 500	26 500	777 000	23 700	26 000	7 040
120/5	23 900	19 800	620 000	18 100	22 200	5 720
180/0	40 900	29 700	1 010 000	39 100	37 400	10 800
180/5	18 900	14 900	481 000	23 900	23 100	6 640
240/0	24 400	19 300	623 000	30 100	32 200	8 820
240/5	12 400	7 060	285 000	29 500	20 200	7 150

## Data Availability

Data available in Mater-Bi/brewers’ spent grain biocomposites—novel approach to plant-based waste filler treatment by highly efficient thermomechanical and chemical methods.

## Supplementary Materials

The following supporting information can be downloaded at: https://www.mdpi.com/article/10.3390/ma15207099/s1, Figure S1: Plots of storage modulus and loss modulus vs. angular frequency for pure Mater-Bi and Mater-Bi composites filled with BSG subjected to various thermomechanical and chemical treatments.
