# Supplementary material for: Mater-Bi/Brewers’ Spent Grain Biocomposites—Novel Approach to Plant-Based Waste Filler Treatment by Highly Efficient Thermomechanical and Chemical Methods
Source: Materials (Basel). 2022 Oct 12;15(20):7099. doi: 10.3390/ma15207099 (PMC9605683; doi:10.3390/ma15207099)
Supplement: Supplementary file 1 [file materials-15-07099-s001.zip › materials-1934170-supplementary.pdf]

materials

MDPI

Supplementary Materials

Mater-Bi/Brewers' Spent Grain Biocomposites—Novel Approach to Plant-Based Waste Filler Treatment by Highly Efficient Thermomechanical and Chemical Methods

Aleksander Hejna [1,2,*], Mateusz Barczewski [1], Paulina Kosmela [2], Olga Mysiukiewicz [1], Paweł Sulima [3], Jerzy Andrzej Przyborowski [3] and Daria Kowalkowska-Zedler [4]

1 Institute of Materials Technology, Poznan University of Technology, Piotrowo 3, 61-138 Poznań, Poland
2 Department of Polymer Technology, Gdańsk University of Technology, Narutowicza 11/12 80-233 Gdańsk, Poland
3 Department of Genetics, Plant Breeding and Bioresource Engineering, University of Warmia and Mazury in Olsztyn, Plac Łódzki 3, 10-724 Olsztyn, Poland
4 Department of Inorganic Chemistry, Gdańsk University of Technology, Narutowicza 11/12, 80-233 Gdańsk, Poland
* Correspondence: ohejna12@gmail.com

Citation: Hejna, A.; Barczewski, M.; Kosmela, P.; Mysiukiewicz, O.; Sulima, P.; Przyborowski, J.A.; Kowalkowska-Zedler, D. Mater-Bi/Brewers' Spent Grain Biocomposites—Novel Approach to Plant-Based Waste Filler Treatment by Highly Efficient Thermomechanical and Chemical Methods. *Materials* **2022**, *15*, 7099. https://doi.org/10.3390/ma15207099

Academic Editor: Swarup Roy

Received: 08 September 2022
Accepted: 10 October 2022
Published: 12 October 2022

Publisher's Note: MDPI stays neutral with regard to jurisdictional claims in published maps and institutional affiliations.

Copyright: © 2022 by the authors. Submitted for possible open access publication under the terms and conditions of the Creative Commons Attribution (CC BY) license (https://creativecommons.org/licenses/by/4.0/).

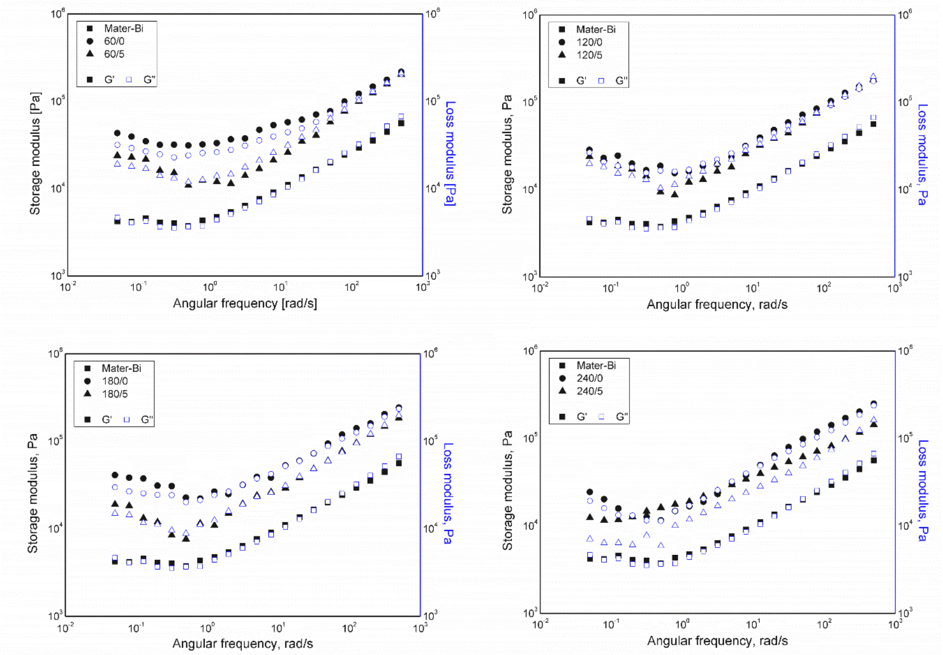

Figure S1. Plots of storage modulus and loss modulus vs. angular frequency for pure Mater-Bi and Mater-Bi composites filled with BSG subjected to various thermomechanical and chemical treatments.
